# Integrating Computational Analysis of In Vivo Investigation of Modulatory Effect of *Fagonia cretica* Plant Extract on Letrozole-Induced Polycystic Ovary Syndrome in Female Rats

**DOI:** 10.3390/biology14070903

**Published:** 2025-07-21

**Authors:** Ayesha Qasim, Hiram Calvo, Jesús Jaime Moreno Escobar, Zia-ud-din Akhtar

**Affiliations:** 1 Centro de Investigación en Computación, Instituto Politécnico Nacional, Ciudad de México 07700, Mexico; qasim23@cic.ipn.mx (A.Q.); jmorenoe@cic.ipn.mx (J.J.M.E.); akhtar23@cic.ipn.mx (Z.-u.-d.A.); 2 University College of Pharmacy, University of the Punjab, Lahore 54590, Pakistan

**Keywords:** *Fagonia cretica*, PCOS, letrozole induced PCOS, modulatory effect, in vivo investigation, hydro-alcoholic, female rats

## Abstract

*Fagonia cretica*, a medicinal herb from the Zygophyllaceae family, is traditionally utilized to treat various conditions such as hepatitis, gynecological disorders, tumors, urinary tract issues, and diabetes. The present study aimed to evaluate the therapeutic potential of *Fagonia cretica* in treating polycystic ovarian syndrome (PCOS) induced in female rats. PCOS, a complex hormonal disorder, was experimentally induced by administering Letrozole (1 mg/kg) in combination with a high-fat diet for 21 days. The affected rats were then treated with hydro-alcoholic extracts of *Fagonia cretica* at doses of 100 mg/kg, 200 mg/kg, and 300 mg/kg for 20 days. Key biochemical parameters—including serum testosterone, insulin, fasting blood glucose, insulin resistance (HOMA-IR), cholesterol, triglycerides, and lipoprotein levels—were measured. Ultrasound imaging and histopathological analysis of ovarian tissues were also performed. The data were analyzed using computer-based statistical tools, including one-way ANOVA, Cohen’s d effect size, and Tukey’s HSD test, with graphical representations generated using Python 3.10 on the Kaggle platform. Results demonstrated a significant reduction in serum testosterone, insulin, cholesterol, and triglyceride levels (*p* < 0.05) in treated groups, along with improved ovarian morphology. These findings support the therapeutic potential of *Fagonia cretica* as a natural treatment for PCOS.

## Simple Summary

Polycystic ovarian syndrome is a common condition in women that affects hormones, weight, and fertility. It can also increase the risk of diabetes and heart problems. This study looked at whether a natural plant called *Fagonia cretica*, traditionally used in herbal medicine, could help improve symptoms of this condition. We used female rats that were given a special diet and medicine to create similar symptoms. Then, we treated them with different amounts of the plant extract. To carefully study the effects, we used computer tools and programming to analyze the results and create clear visual comparisons. The results showed that the plant extract helped reduce hormone levels, blood sugar, and unhealthy fats, while also improving the condition of the ovaries. The strongest improvements were seen at the highest dose of the extract. This research suggests that *Fagonia cretica* could be a useful natural option for managing this condition, and it shows how combining traditional knowledge with modern technology can lead to valuable discoveries in health.

## 1. Introduction

Phytotherapy, or the use of medicinal plants, has been practiced for thousands of years and continues to play a critical role in global healthcare, especially in areas with limited access to modern treatments. The World Health Organization recognizes herbal medicine as a vital part of healthcare systems, with more than 21,000 plant species used medicinally around the world [1,2]. This highlights the necessity of evaluating traditional remedies to meet current medical challenges. Among these challenges, endocrine disorders—including diabetes, lipid imbalances, and reproductive hormonal issues—represent a growing global health concern [3].

Plant-based medicines offer an appealing therapeutic alternative due to their lower cost and reduced side effects. Over 400 plant species are known to support blood sugar regulation [3,4]. Notable examples include *Allium sativum*, *Aloe barbadensis*, and *Fagonia cretica*, the latter of which is traditionally used to treat gynecological disorders, metabolic conditions, and infections [4]. Similarly, herbal treatments have been extensively used for reproductive endocrine disorders, such as amenorrhea and polycystic ovary syndrome (PCOS), with traditional Persian medicine alone documenting over 87 herbs for menstrual regulation [5].

PCOS, previously termed Stein-Leventhal syndrome, is a complex and prevalent disorder affecting reproductive-age women. It is marked by disruptions in the hypothalamus–pituitary–ovarian axis, leading to excessive androgen production, ovarian cyst formation, irregular menstruation, and metabolic disturbances [6,7]. According to the World Health Organization, PCOS is a group II ovulation disorder, characterized by hyperandrogenism, ovarian dysfunction, and polycystic ovarian morphology [8,9]. The hormonal imbalance typically involves elevated levels of luteinizing hormone (LH), which overstimulates ovarian theca cells to produce excessive androgens, thereby interfering with follicular development and ovulation [10,11].

PCOS affects an estimated 4% to 10% of women globally, with significantly higher rates reported in South Asian populations. In Pakistan, prevalence ranges between 15.7% and 37%, while developed countries like the United States report a prevalence of 5–10%, affecting approximately 6.8 million women [11]. Despite its widespread occurrence, the exact cause of PCOS remains unclear, involving a complex interplay of genetic, hormonal, and environmental factors [12,13].

*Fagonia cretica*, a drought-resistant plant found in regions spanning from North Africa to South Asia, has long been used in folk medicine to treat a wide range of ailments, including tumors, infections, liver disorders, and menstrual irregularities [14,15,16]. Its pharmacological effects are attributed to a diverse array of bioactive compounds such as flavonoids, saponins, alkaloids, and sterols [17,18]. Scientific studies suggest that its extracts possess anti-inflammatory, antioxidant, antitumor, and antidiabetic properties [17,18,19]. Recent research also indicates that compounds like rutin and other flavonoids in *Fagonia cretica* may play a role in regulating reproductive hormones and metabolic processes relevant to PCOS [20,21,22].

Despite these promising properties, few studies have specifically investigated the plant’s effect on hormonal and metabolic profiles in PCOS. This study aims to evaluate the therapeutic potential of *Fagonia cretica* using a well-established rat model of PCOS induced by Letrozole, a compound known to mimic the hormonal imbalances seen in the disorder [23]. Through an integrative approach combining experimental pharmacology, histopathological assessment, and computer-based statistical analysis using one-way ANOVA and Cohen’s d, the study explores the modulatory effects of different doses of *Fagonia cretica* extract on key biochemical and hormonal parameters.

The principal findings demonstrate significant improvements in insulin sensitivity, blood lipid levels, and ovarian morphology, especially at higher doses of the extract. These results contribute to the growing body of evidence supporting plant-based interventions for endocrine disorders and highlight the therapeutic promise of *Fagonia cretica* in managing PCOS.

## 2. Materials and Methods

### 2.1. Chemicals and Instruments

The study utilized various chemicals and instruments essential for experimental procedures. The chemicals included Chloroform and Tween 20 (Sigma-Aldrich, St. Louis, MI, USA), Ketamine (MTI-Medical Pvt. Ltd., Lahore, Pakistan), Letrozole (Pacific Pharmaceuticals, Lahore, Pakistan), Methanol (Daejung KOSDAQ, Siheung-si, Republic of Korea), Normal Saline (MediPak Limited, Lahore, Pakistan), and Xylazine (Mylab Pvt. Ltd., Lahore, Pakistan). The instruments employed were as follows: an analytical balance (AB54-S, Mettler Toledo, Greifensee, Switzerland), chiller (MDF-U32V, SANYO Electric Co., Ltd., Osaka, Japan), rat weighing balance (SF-400A, AWR Smith, Randburg, South Africa), refrigerated centrifuge machine (2-16PK, Sigma Laboratories, Schnelldorf, Germany), rotary evaporator (Heidolph Laborota 4002, Sigma-Aldrich, Darmstadt, Germany), universal oven (Model U30, Memmert, Schwabach, Germany), UV-Vis spectrophotometer (UV-2500 PharmaSpec, Shimadzu Corp., Kyoto, Japan), and ultrasound machine (Esoate, Genoa, Italy).

### 2.2. Housing of Animals

Adult healthy Female Sprague Dawley rats of weight ranging from 182 g to 312 g were attained from the animal house of the Punjab University College of Pharmacy, Lahore. The rats were kept under a controlled temperature of 25 ± 2 °C and relative humidity of 60. The rats were maintained in a 12 h/12 h light and dark cycle, were housed in transparent plastic cages with steel coverings, and given a standard diet and water ad libitum. Animals were handled and looked after in accordance with the guidelines set up by the Animal Ethics Committee of Punjab University College of Pharmacy. All experimental protocols were also approved by the Animal Ethics Committee of Punjab University College of Pharmacy. The approval number of study design from Animal Ethics Committee is D/395/IIM.

### 2.3. Collection of Plant

The powder of the *Fagonia cretica* plant was received from Dr. Ruksana, Assistant Professor, Punjab University College of Pharmacy, Lahore, Pakistan. *Fagonia cretica* was identified by Dr. Uzma Hnaif, Assistant Professor at the Department of Botany, Government College University Lahore, Pakistan. She registered the voucher number GC. Herb. Bot. 1929 for this experimental protocol. After taking the plant powder, the research study was proceeded by following steps:

- Extract preparation.
- Characterization of plant extract.
- Phytochemical analysis of extract.
- Designing the experimental protocol.

### 2.4. Preparation of Hydro-Alcoholic Extract

A total of 350 g of powder sample was soaked in 1000 mL of hydro-alcoholic solvent (30:70) in a 1.5 L conical flask; the opening of flask was closed with a stopper to prevent evaporation of the alcohol (methanol). The soaked powder was vigorously shaken at first and then was kept soaking for 3 days with frequent shaking from time to time. After 3 days of soaking, the filtrate was filtered from the powder using Whatman’s filter paper No. 1. The collected filtrate from the first batch of soaking was evaporated in a Heidolph rotary machine in order to evaporate solvent and to make the extract purer.

After running the filtrate of the first batch of soaking through the rotary machine, the evaporated solvent (hydro-alcoholic) was recovered and was used to soak the filtered cake of *Fagonia* powder for the second time and the process of rotary was repeated. The purified extract that was obtained from both the first and second batch was mixed and kept in an oven at 37 °C to dry the remaining solvent (hydro-alcoholic) until a semisolid consistency was obtained. The weight of extract obtained was measured, and the percentage yield was calculated using the following formula:

% yield = (wt. of powder − wt. of extract)/wt. of powder(1)

### 2.5. Characterization of Plant Extract

#### 2.5.1. Qualitative Phytochemical Analysis Test

In order to find out the presence of constituents (Table 1) in *Fagonia cretica* qualitatively, the following tests were performed [24].

#### 2.5.2. Total Phenolic Content (TPC)

To assess the total phenolic content in a hydro-alcoholic plant extract, a small volume of the extract (40 µL), diluted in methanol to a concentration of 3 mg per 10 mL, is mixed with distilled water (3.16 mL) and Folin–Ciocalteu reagent (200 µL). After an 8 min resting period, sodium carbonate (600 µL) is added. Following a half-hour incubation at 40 °C, the absorbance is read at 765 nm. Phenolic content is calculated as mg of gallic acid equivalents per gram of extract, based on a standard gallic acid curve [25].

#### 2.5.3. Total Flavanoid Content (TFC)

To determine the Total Flavonoid Content of a hydro-alcoholic plant extract, begin by mixing 0.3 mL of the extract (containing 3 mg extract per 10 mL methanol) with 3.4 mL of 30% methanol–water solution. Add 150 µL of 0.5 M sodium nitrite, wait for 5 min, then add 150 µL of 0.3 M aluminum chloride. After another 5 min interval, add 1 mL of 1 M sodium hydroxide. Once the solution is clear, measure the absorbance at 506 nm and then estimate the TFC as micrograms of rutin equivalents (RE) per gram of the extract using a rutin calibration curve [25].

### 2.6. Experimental Protocol

Fifteen healthy female rats were indiscriminately allocated into three groups as follows:

(a) Control group (*n* = 3):

This group contained 3 female rats. These rats were given a regular diet with water and 10% of Tween 20 as a solvent till end of the study.

(b) Disease group (*n* = 3):

This disease group contained 3 female rats. These rats were given 1 mg/kg of Letrozole by oral gavage with a high fat diet for 21 days for the induction of polycystic ovarian syndrome [26].

(c) Treatment groups (*n* = 9):

This group was divided into 3 subgroups.

Group 1: 100 mg/kg dose of hydro-alcoholic extract of *Fagonia cretica* (*n* = 3): This group contained 3 female rats. After induction of PCOS each rat is given 100 mg/kg dose of the extract for 20 days.

Group 2: 200 mg/kg dose of hydro-alcoholic extract of *Fagonia cretica* (*n* = 3): This group contained 3 female rats. After induction of PCOS each rat is given 200 mg/kg dose of the extract for 20 days.

Group 3: 300 mg/kg dose of hydro-alcoholic extract of *Fagonia cretica* (*n* = 3): This group contained 3 female rats After induction of PCOS each rat is given 300 mg/kg dose of the extract for 20 days.

### 2.7. Ultrasound Examination of Polycystic Rat

In order to confirm the development of PCOS, after the completion of 21 days of Letrozole 1 mg/kg by oral gavage, all the rats were subjected to ultrasound for the diagnosis of cyst development in ovaries. The ultrasound examination was performed at the University of Veterinary and Animal Sciences, Lahore.

### 2.8. Collection of Blood

The rats were anesthetized with choloroform and blood samples were taken by cardiac puncture. The blood was centrifuged at 2000 rpm for 15 min and serum was prepared and stored at −20 °C for further experiments.

### 2.9. Biochemical Analysis

#### 2.9.1. Serum Hormone Levels (Testosterone and Insulin)

For the evaluation of testosterone and serum insulin levels, blood samples were sent to Zeenat Laboratories, Pakistan. They used HPLC/MS/MS System for evaluating the testosterone level and C-peptide test for measuring the serum insulin level.

#### 2.9.2. Fasting Blood Glucose

For the measurement of serum fasting blood glucose, an On Call Plus glucometer (ACON Laboratories Inc., San Diego, CA, USA) was used.

#### 2.9.3. Insulin Resistance by HOMA-IR Index

HOMA-IR index is one of the methods by which insulin resistance can be determined using two parameters. Fasting insulin levels and fasting glucose levels values are incorporated in the following formula:

Insulin (mU/L) × Glucose (mmol/L) ÷ 22.5(2)

#### 2.9.4. Lipid Profile

Serum triglycerides, serum cholesterol, and serum low density lipoprotein were measured by taking absorption with the help of UV Spectrophotometer (Shimadzu Corporation, Kyoto, Japan) by using TERSACO commercial kit (TERSACO Diagnostics, Lahore, Pakistan). Serum high density lipoprotein was measured by using a BioScience commercial diagnostic kit (BioScience Diagnostics, Karachi, Pakistan) according to manufacturer’s guidelines.

### 2.10. Histopathological Evaluation

The dissected ovary samples were preserved in 10% formalin. The samples were then fixated in the fixation solution for 5 days. After completing of five days, the fixative solution was then rinsed off by running tap water overnight. The wet samples were dried by dehydrating the samples in different concentrations of alcohol. First the samples were placed in 70% alcohol for 7 h. Then in 85 and 95% alcohol for 4 h, then in absolute alcohol for 1 h each. Finally, they were places in a mixture of alcohol and xylene for 45 min. The dehydrating solutions were then cleared from the ovarian tissue by placing the sample in xylene for 30 min. The samples were then positioned in melted paraffin for 2 h for the purpose of infiltration. The infiltrated tissues were then embedded in paraffin and set in plastic cast in the shape of blocks. The sample tissues were then cut into thin slices with a microtome. These sections were flattened by floating them in a water bath at 50–55 °C. The flat sections were then mounted on the slide. Mounting the tissue on a slide was performed by dipping the slides under the floating sections, allowing the sections to adhere to the slides. The slides with mounted sections were dried at 37–40 °C for 2–3 h, then incubated at 50 ± 5 °C for 30–60 min. Finally, the slides were stained using Hematoxylin and Eosin (H&E) staining. After staining, the slides were placed in xylene, absolute alcohol, and 70% alcohol for 3 min each. Slides were washed for 5 min and then dipped in acid alcohol 2–5 times, rinsed again with water for 3 min, then subjected to ammonia alcohol for 3 min, and again rinsed with water. A second stain, Eosin Y, was poured onto the slides and the whole process of drying and rinsing was repeated. A glass cover was placed on sections of tissue. A research microscope was used for examination of the slides at the magnification of 10 × 10.

### 2.11. Statistical and Graphical Analysis

The data were analyzed using one-way ANOVA, Cohen’s d effect size, and Tukey HSD post hoc tests. Statistical computation and graphical representations (bar plots, comparison graphs) were performed using Python 3.10 programming language via the Kaggle cloud-based platform, leveraging various libraries.

## 3. Results

### 3.1. Percentage Yield of Extract

The percentage yield of aqueous alcoholic extract prepared was calculated according to the following formula:

% yield = ((wt. of powder − wt. of extract)/wt. of powder) × 100

Percentage yield = 6.8%

### 3.2. Total Phenolic Content (TPC)

The Total Phenolic Content (TPC) of the hydro-alcoholic extract of *Fagonia cretica* was measured and found to be 103.37 mg GAE (gallic acid equivalents).

### 3.3. Total Flavonoid Content (TFC)

The Total Flavonoid Content (TFC) of the hydro-alcoholic extract of *Fagonia cretica* was measured and calculated to be 76 mg RE/g extract.

### 3.4. Qualitative Phytochemical Analysis

The following tests were performed for phytochemical analysis (Table 2) of hydro-alcoholic extract of *Fagonia cretica* plant:

### 3.5. Biochemical and Statistical Analysis

**Figure 1:**
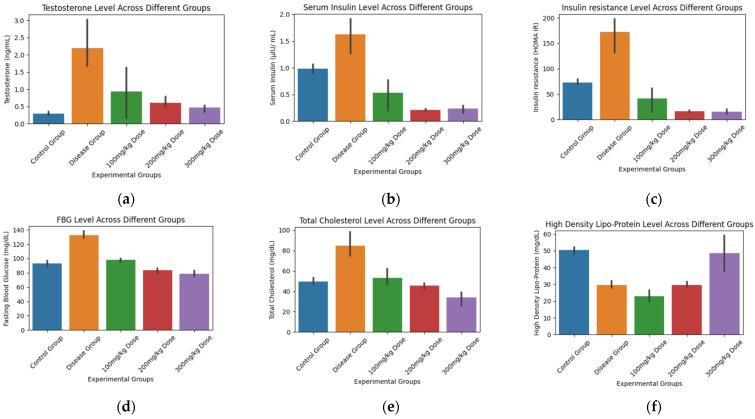

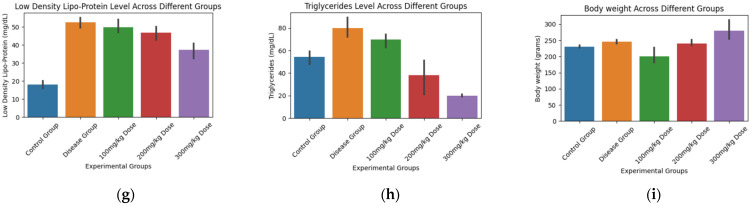
Graphical representation of (**a**) Testosterone Level, (**b**) Serum Insulin, (**c**) Insulin Resistance, (**d**) Fasting Blood Glucose, (**e**) Total Cholesterol, (**f**) HDLP, (**g**) LDLP, (**h**) TG, and (**i**) BW among different experimental groups.

We examined the impact of *Fagonia cretica* plant extract at varying doses on a range of biochemical analysis tests. To visually represent our findings (Figure 1), bar plots were constructed. Additionally, statistical analysis was made using one-way ANOVA (Table 3), Cohen’s d effect size, and Tukey HSD post hoc tests (Table 4).

### 3.6. Ultrasound Examination for Diagnosis of PCOS

#### 3.6.1. Ultrasound Examination of Female Rats of Control Group

**Figure 2:**
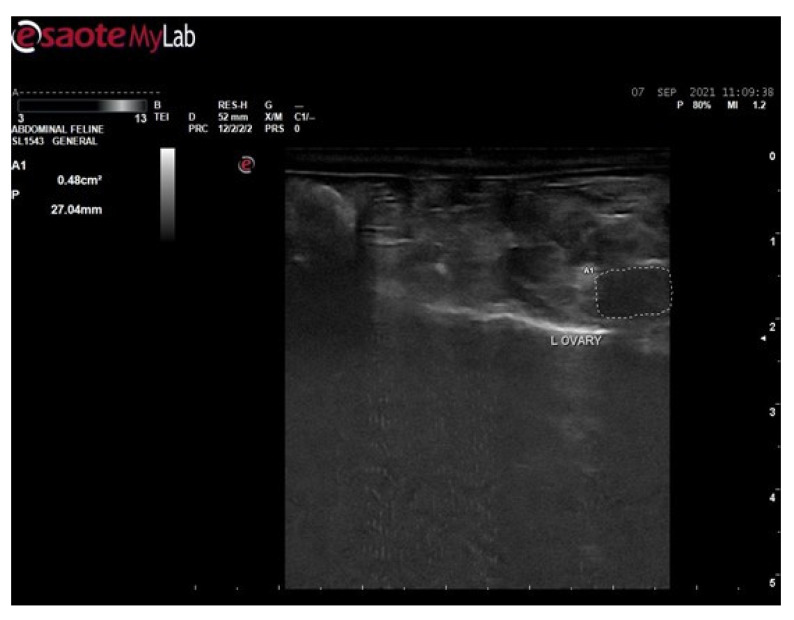
Ultrasound examination of female rats of control group.

The ultrasound examination of the control group (Figure 2) was performed after giving 0.5 mL of 10% Tween 20 for 21 days; the results showed that there were no visible cysts present and the size of the ovary was 27.04 mm or 0.48 cm^2^ as shown in the figure below:

#### 3.6.2. Ultrasound Examination of Female Rats of Disease Group

**Figure 3:**
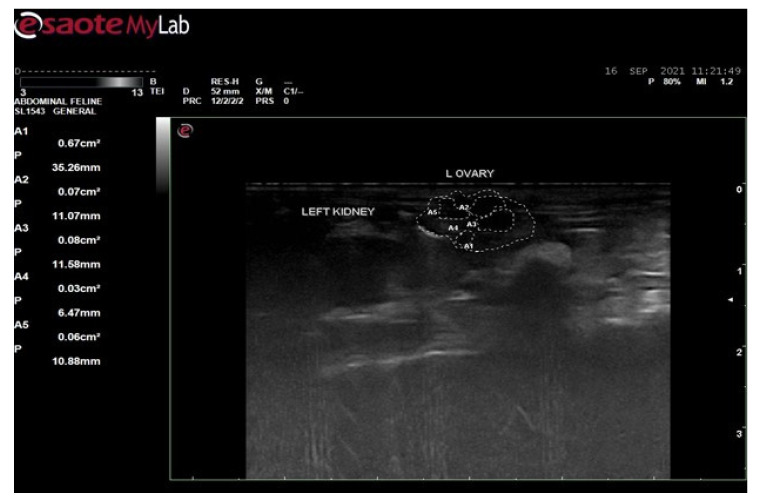
Ultrasound examination of female rats of disease group.

Ultrasound examination of female rats of disease group (Figure 3) was performed to confirm the induction of polycystic ovarian syndrome after administration of 1 mg/kg body weight dose of Letrozole for 21 days. The results showed that the size of the ovary was larger due to the formation of cysts, i.e., 0.67 cm^2^ or 35.26 mm. The formation of cysts were observed as black holes. A total of 4 black holes, the area of which is labeled as A2, A3, A4 and A5 were observed. The sizes of the cysts were as follows: A2 = 0.07 cm^2^, A3 = 0.08 cm^2^, A4 = 0.03 cm^2^, and A5 = 0.06 cm^2^.

### 3.7. Histopathological Evaluation

#### 3.7.1. Histopathology of Ovary of Female Rats of Control Group

**Figure 4:**
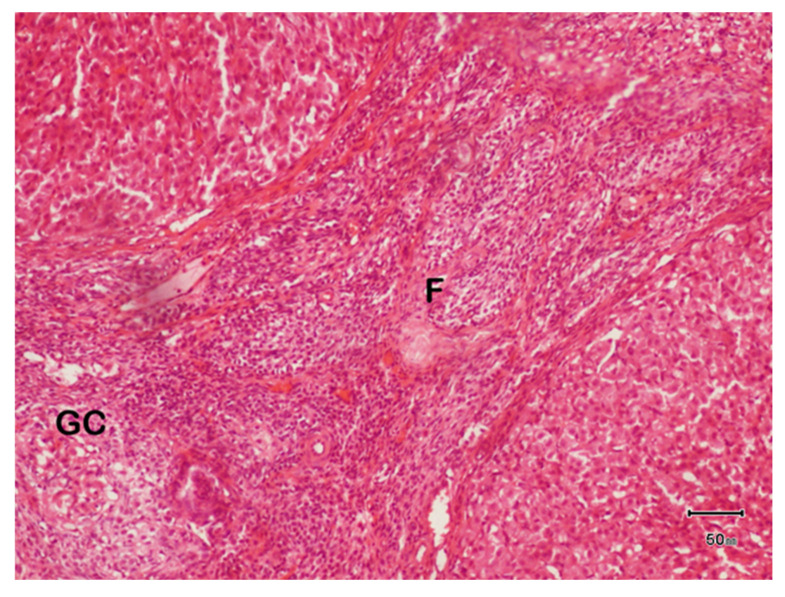
Light photomicrograph of female ovarian tissue of control group.

Histopathology slide showed the histology of the left ovary of a female rat of control group (Figure 4). Ovarian size was 6 mm and ovarian color was grayish. There were no structural abnormalities.

The image was taken at the magnification of 10 × 10. The number of layers of granulosa cells were 11, denoted as GC. The number of follicles observed was 14, denoted as F and an arrow. The number of cysts was zero.

#### 3.7.2. Histopathology of Ovary of Female Rat of Disease Group

**Figure 5:**
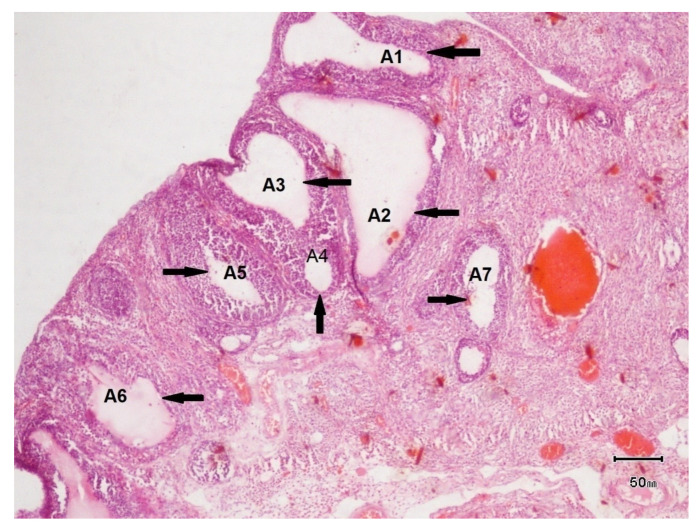
Light photomicrograph of female ovarian tissue of disease group cysts are denoted by arrow heads and labeled as A1, A2, A3, A4, A5, A6, and A7. The number of follicles was 22. The number of cysts was 7.

The histopathological evaluation of the female rats of disease group (Figure 5) showed a visible formation of cysts. The ovarian size was 10 mm, greater than that of control group. The ovarian color was grayish. Structural abnormalities were present.

#### 3.7.3. Histopathology of Ovaries of Female Rat Treated with *F. cretica* Plant Extract

(a) 100 mg/kg dose of hydro-alcoholic plant extract of *F. cretica*

**Figure 6:**
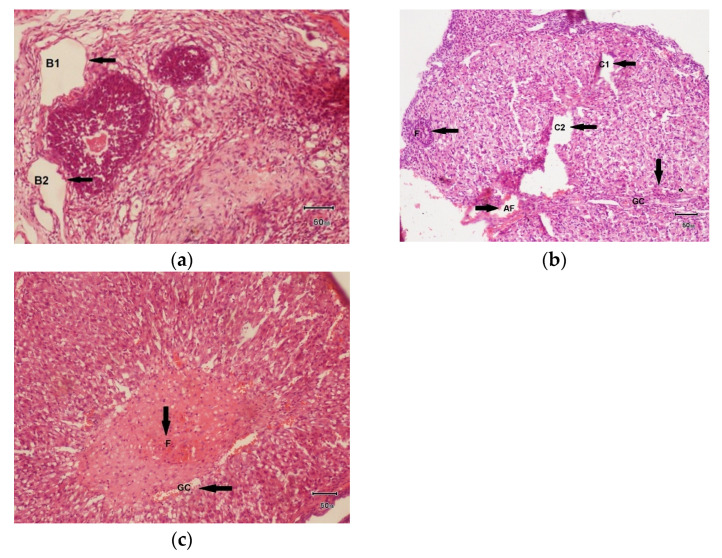
(**a**) 100 mg/kg: H&E-stained light photomicrograph of ovarian tissue of a female rat treated with hydro-alcoholic extract of *Fagonia cretica* at 100 mg/kg dose. Cysts are denoted as B1 and B2. (**b**) 200 mg/kg: The number of cysts observed were 2, denoted by C1 and C2. The number of follicles present were 10, denoted by F. AF represents atretic follicles. The ovarian size was 6 mm. A total of 25 layers of granulosa cells were present, denoted by GC. (**c**) 300 mg/kg: The number of follicles were 16 denoted by F. The size of the ovary was 6 mm. There were 11 layers of granulosa cells, denoted by GC.

The histopathological examination of the sample of ovaries of female rats treated with FCPE at a dose of 100 mg/kg (Figure 6a) showed healing of ovarian tissue and a decreased number of cysts as compared to the disease group. Only 2 cysts were found, which are denoted as B1 and B2. The size of the ovary was 10 mm. The ovarian color was grayish. Only some corpora lutea were seen.

(b) 200 mg/kg dose of hydro-alcoholic extract of *Fagonia cretica*

The histopathological examination of the sample of the left ovary of female rats treated with hydro-alcoholic extract of *Fagonia cretica* at a dose of 200 mg/kg (Figure 6b) showed visible healing of ovarian tissues and the number of cysts decreased even more as compared to that of the 100 mg/kg dose.

(c) 300 mg/kg dose of hydro-alcoholic extract of *Fagonia cretica*

The histopathological examination of the sample of the left ovary of the female rat treated with hydro-alcoholic extract of *Fagonia cretica* at a dose of 300 mg/kg (Figure 6c) showed visible healing of ovarian tissues and the number of cysts was zero.

## 4. Discussion

Polycystic Ovarian Syndrome is a complex endocrine disorder affecting approximately 4–10% of women, as estimated by the National Institute of Health [27]. Characterized by ovarian cysts and excessive androgen production, PCOS manifests in various symptoms, including acne, hirsutism, irregular menstruation, and unexplained weight gain, often leading to obesity [28]. These symptoms stem from a complex interplay of factors like hyperandrogenism, insulin resistance, lifestyle choices, and environmental stressors. Research indicates a strong correlation between Type 1 diabetes and PCOS, with studies suggesting that at least 25% of women with Type 1 diabetes, particularly those using exogenous insulin, are likely to develop PCOS [29]. The multifaceted nature of PCOS, with its diverse clinical presentations, poses a significant challenge for treatment. Addressing all symptoms and underlying causes with a single allopathic approach is often difficult, leading to polypharmacy and the potential for adverse effects from long-term medication use. For instance, prolonged metformin therapy for ovulation induction can result in a vitamin B12 deficiency [30].

The limitations of conventional PCOS treatments, particularly the heightened risks during pregnancy, have spurred interest in safer, plant-based alternatives. Given the link between diabetes and PCOS, *Fagonia cretica*, a plant traditionally used to manage diabetes, emerges as a promising candidate for investigation.

*Fagonia cretica*, belonging to the Zygophyllaceae family, boasts a rich history in traditional medicine, particularly for ailments like hepatitis, gynecological issues, tumors, urinary, and gastric disorders, notably diabetes. Recent studies highlight its positive impact on metabolic markers associated with obesity, including reduced food consumption, weight gain, and improved lipid profiles. Furthermore, *F. cretica* exhibits dose-dependent hypoglycemic effects, significantly lowering serum glucose levels in animal models.

While *F. cretica* demonstrates promise in addressing obesity, diabetes, lipid disorders, and insulin resistance—all interconnected with PCOS—its direct effects on testosterone levels in PCOS remain unexplored. However, research on flavonoids, a class of compounds found in *F. cretica*, offers compelling evidence. For instance, apigenin, a flavonoid, has been shown to regulate hormonal imbalances in PCOS, reducing testosterone, estrogen, and progesterone levels, improving LH/FSH ratios, and decreasing cyst formation. Notably, *F. cretica* exhibits a total flavonoid content ranging from 30 to 545 mg/L rutin equivalent. Rutin, a citrus flavonoid, has demonstrated protective effects against PCOS by mitigating insulin resistance, activating brown adipose tissue, and improving menstrual cyclicity and fertility [31,32].

These findings strongly support the investigation of *F. cretica* hydro-alcoholic extract as a potential treatment for PCOS. The study employed a Letrozole-induced PCOS model, where a 1 mg/kg body weight dose of Letrozole was administered for 21 days via oral gavage. Letrozole, a non-steroidal aromatase inhibitor, disrupts estrogen production, leading to hyperandrogenism and mimicking the hormonal imbalances characteristic of PCOS.

The statistical analysis of the biochemical tests using one-way ANOVA and Cohen’s d reveals significant differences between the control and treatment groups. The ANOVA results indicate that there are statistically significant differences (*p* < 0.05) in all biochemical tests measured, including testosterone, SI, HOMA IR, FBG, CHOL, TGR, LDLP, HDLP, and BW. The F-values range from 5.09 to 89.58, showing varying degrees of between-group variance. Cohen’s d provides insights into the effect sizes of these differences. For instance, the comparison of the control group with the disease group shows large negative effect sizes, indicating substantial deterioration in the disease group for most tests (e.g., Cohen’s d = −14.06 for LDLP and −8.75 for FBG).

Conversely, certain treatment dosages, particularly those of 200 mg/kg and 300 mg/kg, exhibit substantial positive effect sizes compared to the control group, suggesting significant improvements. Notably, the 300 mg/kg dosage has a Cohen’s d of 8.40 for TGR and 3.59 for FBG, highlighting its effectiveness. The 100 mg/kg dosage also shows a significant improvement in SI and HDLP with Cohen’s d values of 2.12 and 9.62, respectively. These results imply that the 200 mg/kg and 300 mg/kg doses of the plant extract are particularly effective in mitigating the adverse effects of the disease, as evidenced by the statistically significant improvements in multiple biochemical markers. Overall, the data suggest that higher doses of the plant extract could potentially offer a therapeutic benefit, particularly in improving lipid profiles and glucose metabolism.

The multiple comparisons of means using the Tukey HSD test indicate several significant differences between the control, disease, and treatment groups for various biochemical tests. For testosterone, significant improvements are observed with the 200 mg/kg (*p* = 0.04) and 300 mg/kg (*p* = 0.03) doses compared to the disease group, indicating efficacy at these dosages. Serum insulin levels show significant improvement across all treatment groups (100 mg/kg, 200 mg/kg, and 300 mg/kg; *p* < 0.001) compared to the disease group, suggesting that the plant extract effectively enhances insulin regulation. HOMA-IR, FBG, and CHOL levels also significantly improve in all treatment groups (*p* < 0.001), with the 300 mg/kg dose showing the most substantial effect. Triglycerides (TGR) significantly improve at the 200 mg/kg (*p* = 0.0013) and 300 mg/kg (*p* = 0.0001) doses, but not at 100 mg/kg (*p* = 0.6418). Low-Density Lipoprotein (LDLP) shows a significant reduction only at the 300 mg/kg dose (*p* = 0.0018). High-Density Lipoprotein (HDLP) and body weight changes are mixed, with significant changes seen at the 300 mg/kg dose (HDLP: *p* = 0.0078, Body weight: *p* < 0.001). Overall, the plant extract, especially at higher doses, demonstrates significant therapeutic potential in improving several biochemical markers associated with the disease. Significant *p*-values (typically *p* < 0.05) indicate that the observed differences are unlikely due to chance, and therefore, the plant extract shows promise as a treatment option.

Ultrasound examination was performed in order to confirm the induction of disease and, according to ultrasound images, the size of the ovary in the control group was 0.48 cm^2^ and in disease group 0.67 cm^2^. The size of the ovary in disease group was increased due to the formation of cysts. The rats were then treated with 100 mg, 200 mg, and 300 mg per kg body weight of hydro-alcoholic extract of *Fagonia cretica* and the effect of the plant on levels of testosterone, serum insulin, insulin resistance, FBG, lipid profile, and body weight were evaluated.

## 5. Limitations and Future Directions

This study was designed as an exploratory, proof-of-concept investigation to assess the potential protective role of *Fagonia cretica* in a PCOS rat model. While the sample size was limited to three animals per group due to ethical and logistical constraints, this was sufficient for initial biological observations and histopathological analysis. Although no standard PCOS medication was used as a positive control, the study aimed to establish a baseline efficacy of *Fagonia cretica* based on its traditional use and bioactive properties. The 20-day duration followed established models for PCOS induction and treatment in rodents and was adequate for observing short-term therapeutic outcomes. Comprehensive dose–response and toxicity assessments were beyond the intended scope of this study but are recommended for future work. Follow-up studies will focus on expanding the sample size, including standard comparisons, and exploring the long-term efficacy and safety of the plant extract.

## 6. Conclusions

The study evaluated the therapeutic potential of hydro-alcoholic extract of *Fagonia cretica* on rats induced with a disease characterized by increased ovarian size due to cyst formation, confirmed via ultrasound (control group: 0.48 cm^2^; disease group: 0.67 cm^2^). Following treatment with 100 mg, 200 mg, and 300 mg/kg body weight doses, significant improvements were observed in multiple biochemical markers. Testosterone levels significantly decreased with 200 mg and 300 mg/kg doses. Serum insulin levels improved significantly across all treatment groups, indicating enhanced insulin regulation. HOMA-IR, FBG, and cholesterol levels showed substantial improvements with all doses, particularly at 300 mg/kg. Triglycerides and LDLP levels significantly reduced at higher doses, while HDLP showed mixed results. The 300 mg/kg dose also significantly reduced body weight. These findings suggest that *Fagonia cretica* extract, especially at higher doses, effectively ameliorates several pathological parameters associated with the disease, demonstrating its potential as a therapeutic agent. Flavanoids that are present as constituent in *Fagonia cretica* may be responsible for its protective effects in PCOS. Further research is needed to investigate the mechanism by which *Fagonia cretica* lowers reproductive and metabolic hormones level.

## Figures and Tables

**Table 1:** Qualitative phytochemical test.

Sr. No	Constituents Expected	Test Name
**1**	Carbohydrate	Molisch Test
**2**	Flavonoid	Lead Acetate Test
**3**	Glycosides	Legal’s Test
**4**	Phenolic Compounds	Iodine Test
**5**	Phytosterols	Salkowski’s Test
**6**	Proteins and Amino Acids	Ninhydrin Test
**7**	Reducing Sugar	Fehling’s Test
**8**	Saponins	Foam Test
**9**	Tannins	Braymer’s Test
**10**	Terpenoids	Salkowski’s Test

**Table 2:** Results of qualitative phytochemical test.

Sr. No.	Test Name	Outcome
1.	Molisch test	Positive
2.	Lead acetate test	Positive
3.	Legal’s test	Positive
4.	Iodine test	Positive
5.	Salkowski’s test	Negative
6.	Ninhydrin test	Positive
7.	Fehling’s test	Positive
8.	Foam test	Positive
9.	Braymer’s test	Positive
10.	Test for Terpenoids	Negative

**Table 3:** One-way ANOVA vs. Cohen’s d.

Sr. No.	Biochemical Tests	F-Value	*p*-Value	Control vs. Disease	Control vs. 100 mg/kg	Control vs. 200 mg/kg	Control vs. 300 mg/kg
1.	Testosterone	5.09	0.01	−3.79	−0.84	−2.61	−2.28
2.	SI	26.31	2.73 × 10^−5^	−2.73	2.12	14.04	10.34
3.	HOMA IR	34.39	8.08 × 10^−6^	−3.95	1.82	14.37	11.72
4.	FBG	89.58	8.65 × 10^−8^	−8.75	−1.47	2.57	3.59
5.	CHOL	20.34	8.56 × 10^−5^	−4.10	−0.62	1.33	2.90
6.	TGR	22.43	5.57 × 10^−5^	−3.46	−2.65	1.36	8.40
7.	LDLP	51.66	1.20 × 10^−6^	−14.06	10.62	−9.81	−5.85
8.	HDLP	17.43	0.0001	9.775	9.62	11.06	0.24
9.	BW	7.48	0.004	−3.13	1.73	−1.36	−2.30

**Table 4:** Multiple comparison of Means—Tukey’s HSD.

Sr. No.	Biochemical Tests	Group 1	Group 2	Mean Diff	*p*-adj	Reject
1.	Testosterone	Control	Disease	1.91	0.017	True
		Disease	100 mg/kg	1.26	0.13	False
		Disease	200 mg/kg	1.59	0.04	True
		Disease	300 mg/kg	1.73	0.03	True
2.	Serum Insulin	Control	Disease	0.64	0.01	True
		Disease	100 mg/kg	1.09	0.0004	True
		Disease	200 mg/kg	1.41	0.0	True
		Disease	300 mg/kg	1.39	0.0001	True
3.	HOMA-IR Index	Control	Disease	99.45	0.0006	True
		Disease	100 mg/kg	130.50	0.0001	True
		Disease	200 mg/kg	155.49	0.0	True
		Disease	300 mg/kg	156.50	0.0	True
4.	FBG	Control	Disease	39.33	0.0	True
		Disease	100 mg/kg	34.33	0.0	True
		Disease	200 mg/kg	48.66	0.0	True
		Disease	300 mg/kg	54.0	0.0	True
5.	CHOL (Cholesterol)	Control	Disease	35.33	0.001	True
		Disease	100 mg/kg	31.66	0.0025	True
		Disease	200 mg/kg	39.43	0.0004	True
		Disease	300 mg/kg	51.00	0.0001	True
6.	TGR (Triglycerides)	Control	Disease	25.33	0.0338	True
		Disease	100 mg/kg	10.0	0.6418	False
		Disease	200 mg/kg	41.33	0.0013	True
		Disease	300 mg/kg	59.66	0.0001	True
7.	LDLP (Low-Density Lipoprotein)	Control	Disease	34.7	0.0	True
		Disease	100 mg/kg	2.93	0.8216	False
		Disease	200 mg/kg	5.9	0.2779	False
		Disease	300 mg/kg	15.26	0.0018	True
8.	HDLP (High-Density Lipoprotein)	Control	Disease	−20.83	0.0041	True
		Disease	100 mg/kg	6.66	0.5394	False
		Disease	200 mg/kg	0.03	1.0	False
		Disease	300 mg/kg	−19.0	0.0078	True
9.	Body Weight	Control	Disease	15.0	0.839	False
		Disease	100 mg/kg	45.0	0.0702	False
		Disease	200 mg/kg	5.0	0.9966	False
		Disease	300 mg/kg	−33.66	0.0	True

## Data Availability

The Python code used for statistical analysis and result generation, including biochemical data processing, has been submitted as a supplementary file titled “fagonia_python_code.ipynb” and can be accessed via platforms such as Kaggle or Google Colab.

## Supplementary Materials

The following supporting information can be downloaded at: https://www.mdpi.com/article/10.3390/biology14070903/s1. Statistical_analysis.
